# Predictors of Plasma Fluoride Concentrations in Children and Adolescents

**DOI:** 10.3390/ijerph17249205

**Published:** 2020-12-09

**Authors:** Jenny L. Carwile, Katherine A. Ahrens, Shravanthi M. Seshasayee, Bruce Lanphear, Abby F. Fleisch

**Affiliations:** 1 Center for Outcomes Research and Evaluation, Maine Medical Center Research Institute, Portland, ME 04101, USA; sseshasaye@mmc.org (S.M.S.); afleisch@mmc.org (A.F.F.); 2 Muskie School of Public Service, University of Southern Maine, Portland, ME 04101, USA; katherine.ahrens@maine.edu; 3 Faculty of Health Sciences, Simon Fraser University, Vancouver, BC V5A 1S6, Canada; bruce_lanphear@sfu.ca; 4 Pediatric Endocrinology and Diabetes, Maine Medical Center, Portland, ME 04101, USA

**Keywords:** fluoride, tea, water, children, What We Eat in America, National Health and Nutrition Examination Survey

## Abstract

Despite increasing concerns about neurotoxicity of fluoride in children, sources of fluoride exposure apart from municipal water fluoridation are poorly understood. We aimed to describe the associations of demographics, drinking water characteristics, diet, and oral health behaviors with plasma fluoride concentrations in U.S. children. We used data from 3928 6–19-year-olds from the 2013–2016 National Health and Nutrition Examination Survey. We used a 24-h dietary recall to estimate recent consumption of fluoridated tap water and select foods. We estimated the associations of fluoridated tap water, time of last dental visit, use of toothpaste, and frequency of daily tooth brushing with plasma fluoride concentrations. The participants who consumed fluoridated (≥0.7 mg/L) tap water (n = 560, 16%) versus those who did not had 36% (95% CI: 22, 51) higher plasma fluoride. Children who drank black or green tea (n = 503, 13%) had 42% higher plasma fluoride concentrations (95% CI: 27, 58) than non-tea drinkers. The intake of other foods and oral health behaviors were not associated with plasma fluoride concentrations. The consumption of fluoridated tap water and tea substantially increases plasma fluoride concentrations in children. Quantifying the contribution of diet and other sources of fluoride is critical to establishing safe target levels for municipal water fluoridation.

## 1. Introduction

Excess fluoride intake during tooth enamel formation can result in dental fluorosis, a permanent discoloring of the tooth enamel [1]. Numerous studies also indicate that fluoride exposure during early brain development is associated with cognitive deficits in children [2,3,4,5,6]. Little is known, however, about the contribution of diet and other factors that may increase fluoride intake in U.S. children.

Since 1945, fluoride has been added to municipal water in many U.S. cities to prevent dental caries; however, foods, beverages, and fluoridated toothpaste may also contribute to total fluoride intake in children and adolescents. Fluoride can be found into a wide variety of foods and beverages if the items are manufactured (e.g., cereal, soft drinks) or prepared (e.g., pasta, juice prepared from concentrate, coffee, tea) with fluoridated water [7]. Other mechanisms of fluoride uptake are specific to certain foods and beverages [8]. For example, tea plants preferentially uptake or hyperaccumulate fluoride from the soil [9]. Grapes may be treated with cryolite [10]—a pesticide that decomposes to fluoride ion—and processed meats can have fluoride-rich bone particles introduced during mechanical deboning [11]. The use of fluoridated toothpaste may be another important source of fluoride exposure, particularly for young children who swallow it [8,12].

A contemporary assessment of factors influencing total fluoride intake in children and adolescents is critical for determining optimal levels of municipal water fluoridation, particularly in populations vulnerable to adverse health effects of fluoride exposure. Here, we explore associations of demographics, drinking water, diet, and oral health behaviors with plasma fluoride in a nationally representative sample of 6 to 19-year-old U.S. children and adolescents.

## 2. Materials and Methods

We combined data from the 2013–2014 and 2015–2016 cycles of the National Health and Nutrition Examination Survey (NHANES), a nationally representative survey of civilian, non-institutionalized U.S. adults and children. The participants completed an in-person interview, a 24-h dietary recall interview and a blood draw, and NHANES technicians collected a tap water sample from each participating household. After excluding participants with missing data on plasma fluoride (n = 567) or water fluoride (n = 83), there were 3928 participants 6–19 years old included in our analysis. All participants 7–17 years old gave informed assent, parents or guardians of participants < 18 years gave informed consent, and participants aged 18 and older gave informed consent for themselves. The Maine Medical Center Institutional Review Board determined that this study was not human subject research.

Trained interviewers conducted in-person interviews in English or Spanish at the participants’ homes using a Computer-Assisted Personal Interview system. For participants under 16 years, an adult proxy (a family member or legal guardian, preferably with the most knowledge of the child) reported the participant’s age, sex, race/ethnicity, household income, and family size; participants ≥ 16 years old were interviewed directly. We used the Department of Health and Human Services’ poverty guidelines to calculate the family income to poverty ratio. For our analyses, we categorized the income to poverty ratio as <100%, 100–<200%, or ≥200% [13].

Interviewers administered a 24-h dietary recall to NHANES participants at Mobile Examination Centers using the US Department of Agriculture’s (USDA) computerized and validated Automated Multiple Pass Method [14]. For children 6 to 8 years, an adult proxy provided data on dietary intake with the child’s assistance. Children 9–11 years provided data on their dietary intake with assistance from a proxy; children 12 and older recalled their diet independently. The participants (or proxies) reported the types and amounts of food they consumed during the prior 24 h. The participants were provided with measuring guides (e.g., mugs, bottles, thickness sticks) to aid in reporting the amount of food or beverage consumed. Reported intakes were processed using the USDA’s Food and Nutrient Database for Dietary Studies, which is used to code reported foods and portion sizes and calculate nutrient values [15].

We used published databases of fluoride concentrations in foods and beverages [8,16] to identify categories of foods/beverages with the highest potential to contain fluoride: green/black tea, other tea, coffee, fruit juice, grape juice, grapes/raisins, shellfish, and processed chicken (e.g., chicken patties, nuggets, and tenders). We then used the What We Eat in America (WWEIA) database, a classification scheme of approximately 150 unique food and beverage categories produced specifically for each 2-year NHANES cycle, to map individual food items reported by NHANES participants into the above categories [17]. ‘Green and black tea’ and ‘other tea’ did not have unique WWEIA categories, so we used individual food names to map food items into these categories. In the tea categories, we included hot and iced tea, caffeinated and decaffeinated tea, and brewed, bottled, and instant tea. We chose a priori to combine green and black tea into one category, since these are harvested from the same species of plant [18]. ‘Other teas’ included herbal teas (e.g., chamomile, hibiscus), which are made from other plant species, and tea/lemonade drinks, for which the variety of tea was not specified. For analyses, we categorized participants according to whether they reported consuming any food from a given category in the past 24 h.

NHANES technicians collected water samples from participants’ home tap (primary consumption source or faucet most used for drinking and cooking water) after allowing the water to run for 5–10 s. The technicians shipped the water samples on ice overnight to Georgia Regents University (Augusta, GA, USA) for electrometrical measurement of fluoride concentration using the fluoride-specific electrode [6,19]. Prior to public release of the data, NHANES analysts replaced samples below the limit of detection (LOD) of 0.10 mg/L with the LOD/√2.

The participants self-reported tap water source as community water, well or rainwater, spring, don’t drink tap water, or don’t know, and the amount of tap water they consumed within the past 24 h. We considered a participant to have recent consumption of fluoridated home tap water if they reported consuming tap water in the past 24 h and if their home tap water had a fluoride concentration ≥0.7 mg/L (i.e., the optimal amount of fluoridation currently recommended by the US Public Health Service [1]); otherwise, they were considered to have no recent consumption of fluoridated tap water.

NHANES used a Computer-Assisted Personal Interview system to administer an oral health questionnaire to participants at their homes. An adult proxy completed the interview on behalf of children under 16 years; participants 16 and older completed the interview independently. The participants were asked about the timing of their last dental visit, tooth brushing frequency, and amount of toothpaste used, with response categories of ‘full load’, ‘half load’, ‘pea size’, and ‘smear’. The participants were provided with a picture card to aid in their response to the latter item. Participants younger than 15 years were asked if they were currently using prescription fluoride drops or tablets.

Technicians collected blood samples during the Mobile Examination Center exam. Blood samples were frozen at −20 °C and shipped overnight on dry ice to Georgia Regents University for analysis of fluoride concentrations. Plasma fluoride (µmol/L) was measured using the fluoride-specific electrode with the hexamethyldisiloxane facilitated diffusion method [20]. Prior to public release of the data, NHANES analysts replaced samples below the LOD of 0.25 nmol with the LOD/√2. The half-life of plasma fluoride is around 6 h [21], and similar to spot urine fluoride, it reflects a combination of both current and chronic fluoride intake [22].

We used linear regression to examine associations of demographic, dietary, and oral health characteristics with plasma fluoride. We ran models unadjusted and adjusted for demographics [age (continuous), sex, and race/ethnicity (non-Hispanic white, non-Hispanic Black, Hispanic, Asian, other)]. We then ran fully adjusted models that included additional adjustment for the behavioral factors we found to be associated with plasma fluoride (i.e., consumption of fluoridated tap water and recent green/black tea consumption). We natural log-transformed plasma fluoride to improve normality of the residuals. For ease of interpretation, we exponentiated regression coefficients and reported results as a percent change [% change = (exp(beta) − 1) × 100] in plasma fluoride concentration. We estimated the percentage of variance in plasma fluoride concentrations accounted for by each factor using the formula (SS_res, reduced_ − SS_res, full_)/SS_res, reduced_, where SS_res_ is the residual sum of squares for the full or reduced model [23].

We performed several sensitivity analyses. First, we assessed whether there was a dose-response (on the log scale) on plasma fluoride concentrations for consumption of fluoridated tap water and green/black tea, separately. To do this, we categorized intake into servings (8 oz. cup or 236 g) per day and estimated the linear trend *p*-value for each exposure-plasma fluoride association using fully adjusted regression models. Second, we similarly assessed whether there was a dose-response by tap water fluoride concentration, using tap water fluoride cut points that aligned with policy relevant water fluoride concentrations when possible [1]. We also modeled the association between continuous tap water fluoride and plasma fluoride concentrations. Third, because water that is considered fluoridated (≥0.7 mg/L) by a municipality may be somewhat less fluoridated when it reaches the home, we repeated the analyses for consumption of fluoridated tap water defining fluoridation as >0.5 mg/L rather than 0.7 mg/L. Fourth, although black and green tea leaves are harvested from the same species of plant, black tea leaves are higher in fluoride than green tea leaves [24], due to different processing techniques or use of different varieties of tea. For this reason, we assessed associations for green and black tea, separately, with plasma fluoride, adjusting for intake of the other tea variety. We also individually assessed tea based on preparation method (brewed, bottled or instant) adjusting for intake of tea prepared using other methods. Fifth, we examined the association between intake of green/black tea and fluoridated water on plasma fluoride concentrations only in 16–19-year-old girls because this population overlaps with reproductive age women, in whom prenatal exposure has been associated with neurocognitive deficits in offspring [2,5,25]. Finally, we examined the demographics, diet, and oral health behaviors of green/black tea drinkers versus non-drinkers.

For all analyses, we used day 1 dietary recall sample weights and accounted for the complex study design using the study design variables. We conducted statistical analyses using SAS EG, version 7.1 (SAS Institute, Inc., Cary, NC, USA) and R (Core Team. 2018. R: A language and environment for statistical computing. Vienna Austria: R Foundation for Statistical Computing).

## 3. Results

### 3.1. Population Characteristics

Our study population was 52% boys; 51% were non-Hispanic white children and adolescents, with 38% between the ages of 15–19 years. Seventy-one percent reporting used community water (Table 1). Just over half of the participants (n = 1820; 54%) reported consuming any tap water in the past 24 h; only 16% (n = 560) recently consumed fluoridated tap water. Thirteen percent of the participants reported recent consumption of black (11%) or green (2%) tea (n = 503, 13%). The geometric mean plasma fluoride concentration was 0.36 µmol/L [95% confidence interval (CI): 0.34, 0.38]; 20% (n = 863) of participants had plasma fluoride samples below the LOD.

Median home tap water fluoride was 0.59 mg/L (interquartile range (IQR) 0.20) in 2013–2014 and 0.39 mg/L (IQR 0.42) in 2015–2016, which aligned with a 2015 change in the US guidelines for fluoridation of community water from a target of 0.7–1.2 mg/L to 0.7 mg/L [1]. Overall, 1193 participants (29%) had fluoridated home tap water (≥0.7 mg/L); 11% (n = 421) of water samples were below the LOD.

### 3.2. Associations of Demographics, Drinking Water Characteristics, Diet, and Oral Health Behaviors with Plasma Fluoride Concentration

**Figure 1:**
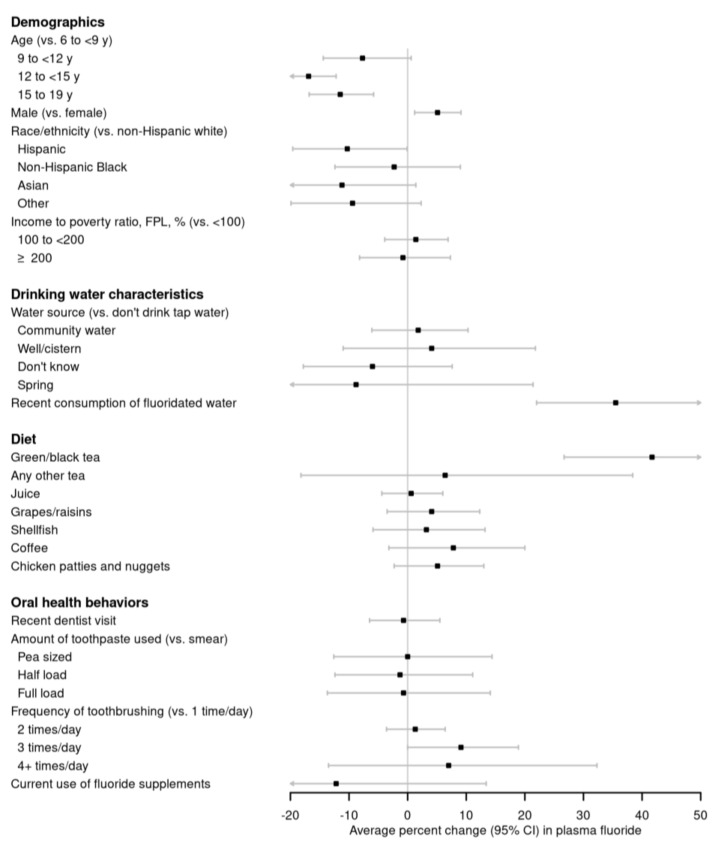
Adjusted percent change (95% confidence interval) in plasma fluoride by demographics, drinking water characteristics, recent diet, and oral health behaviors in 3928 6–19-year-old National Health and Nutrition Examination Survey participants from 2013–2016. Adjusted for age, sex, race/ethnicity, and with mutual adjustment for consumption of fluoridated tap water, and recent green/black tea consumption. See Table A1 for effect estimates and 95% confidence intervals. Abbreviations: CI, confidence interval.

In fully adjusted models, boys (vs. girls) had 5.1% higher plasma fluoride (95% CI: 1.2, 9.1), and Hispanics (vs. non-Hispanic whites) had 10.3% (95% CI: −19.6, −0.1) lower plasma fluoride (Figure 1; Table A1). Relative to 6- to <9-year-old children, older children and adolescents had lower plasma fluoride. Plasma fluoride did not differ by income to poverty ratio. The participants who consumed fluoridated tap water (vs those who did not) had 35.5% (95% CI: 22.0, 50.5) higher plasma fluoride. Participants who reported drinking green/black tea (vs those who did not) had 41.7% (95% CI: 26.7, 58.4) higher plasma fluoride concentrations. Recent consumption of fluoridated tap water accounted for 5% of the variance in plasma fluoride concentrations and green/black tea accounted for 6% of the variance (the overall R^2^ for fully adjusted model = 0.12). Other varieties of tea, other beverages, including coffee and fruit juice, and other culprit foods, including grapes/raisins, chicken nuggets, and shellfish, were not associated with plasma fluoride. Participants who were currently using prescription fluoride drops or tablets had a 12.2% lower (95% CI: −32.0, 13.4) plasma fluoride, although only 2% of the cohort used prescription fluoride drops or tablets and these individuals had lower tap water fluoride concentrations (mean 0.21 mg/L) than those who did not use prescription fluoride products (mean tap water fluoride 0.57 mg/L). Other oral health behaviors, including a recent dentist visit, frequency of tooth brushing, and amount of toothpaste used, were not associated with plasma fluoride. Analyses adjusted for sociodemographic characteristics only (data not shown) were similar to fully adjusted models.

### 3.3. Sensitivity Analyses

**Figure 2:**
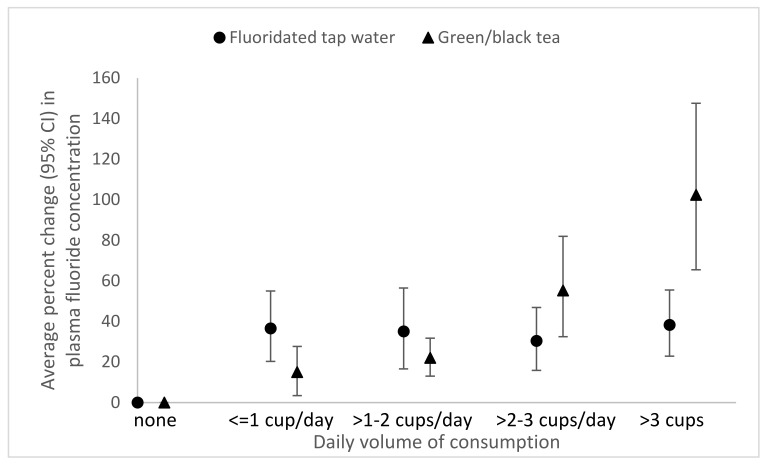
Adjusted percent change (95% confidence interval) in plasma fluoride per number of 8 oz. cups of fluoridated tap water (0 servings/day, n = 3368; >0–1 servings/day, n = 100; >1–2 servings/day, n = 152; >2–3 servings/day, n = 94; >3 servings/day, n = 214) and green/black tea (0 servings/day, n = 3425; >0–1 servings/day, n = 135; >1–2 servings/day, n = 194; >2–3 or more servings/day, n = 78; ≥3 servings/day, n = 96) in 3928 6–19 year old National Health and Nutrition Examination Survey participants from 2013–2016. Adjusted for age, sex, race/ethnicity, fluoridated tap water consumption, and recent green/black tea consumption.

**Figure 3:**
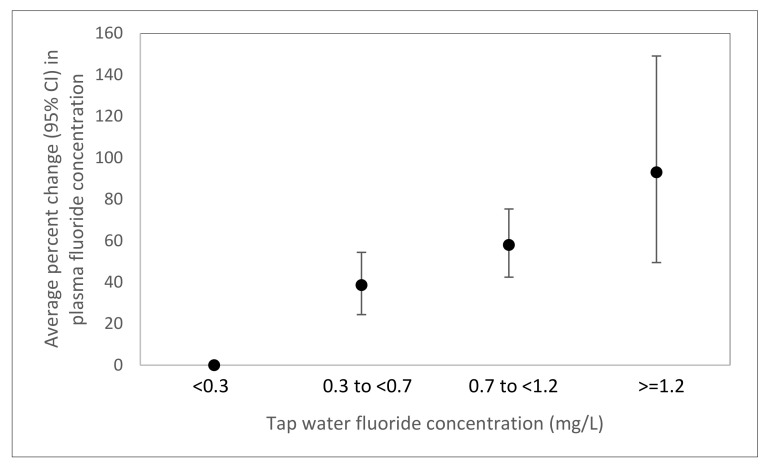
Adjusted percent change (95% confidence interval) in plasma fluoride per fluoride concentration of home tap water (<0.3 mg/L, n = 647; 0.3 to <0.7 mg/L, n = 613; 0.7 to <1.2 mg/L, n = 507; ≥1.2 mg/L, n = 53) in 1820 6–19 year old National Health and Nutrition Examination Survey participants from 2013–2016 who consumed tap water. Adjusted for age, sex, race/ethnicity, and recent green/black tea consumption.

We observed linear dose-responses associations of the amount of fluoridated tap water (*p*-trend < 0.0001) and green/black tea consumed (*p*-trend < 0.0001) with plasma fluoride (Figure 2) as well as the concentration of tap water fluoride and plasma fluoride (*p*-trend < 0.0001) (Figure 3). A 1 mg/L increase in water fluoride concentration was associated with an 83.1% (95% CI: 63.1, 105.5) increase in plasma fluoride.

When we defined water fluoridation as ≥0.5 mg/L, 51% of children had fluoridated tap water, and we found a similar association between fluoridated water consumption and plasma fluoride concentrations (38.7%, 95% CI: 28.3, 50.1), as we did under the original definition of ≥0.7 mg/L (35.5%, 95% CI: 22.0, 50.5).

When we investigated green and black tea separately, we found positive associations of black tea [44.3% higher (95% CI: 27.9, 59.9)] and green tea [24.0% higher (95% CI: 9.0, 51.9] with plasma fluoride concentrations. All methods of tea preparation were associated with plasma fluoride concentrations [for brewed tea, 48.1% higher (95% CI: 27.1, 63.5); for bottled tea; 33.6% higher (95% CI: 14.4, 57.3); for instant tea, 45.9% higher (95% CI: 14.0, 68.7)].

Among 16–19-year-old girls, green/black tea consumption was associated with 64.4% higher (95% CI: 31.7, 105.2) plasma fluoride and fluoridated water consumption was associated with 36.0% higher (95% CI: 17.7, 57.0) plasma fluoride concentrations. When we compared the characteristics of green/black tea drinkers to non-green/black tea drinkers, we found that the prevalence of green/black tea consumption increased with participant age, with recent green/black tea consumption reported by 9% of 6–10 year olds, 14% of 11–15 year olds, and 17% of 16–19 year olds. Green/black tea drinkers were slightly less likely than non-drinkers to have recently consumed fluoridated tap water (10% vs. 14%). Other demographic characteristics were not related to green/black tea intake (Table A2).

## 4. Discussion

In this nationally representative population of 3928 U.S. children 6–19 years old, we found that recent consumption of fluoridated tap water was associated with 36% higher plasma fluoride concentrations, while recent green/black tea consumption was associated with 42% higher plasma fluoride concentrations. Other behaviors were not associated with plasma fluoride concentrations. Consistent with previous observations [26], we observed higher plasma fluoride concentrations, on average, among younger children, boys, and non-Hispanic whites. It is unknown whether these differences reflect unmeasured exposure sources or other factors that affect fluoride metabolism.

Our observed association between recent consumption of fluoridated tap water and plasma fluoride was consistent with that recently reported by the Maternal-Infant Research on Environmental Chemicals (MIREC) study, a Canadian cohort of 1566 women with follow-up beginning during pregnancy [27]. In the MIREC cohort, women living in fluoridated regions had almost double the urinary fluoride concentrations than women in regions without fluoridation (mean ± SD, 1.15 mg/L ± 0.65 vs. 0.60 mg/L ± 0.40), adjusting for demographic characteristics and green/black tea consumption [27]. It is possible that, despite similarities in water fluoride concentrations between the studies, the MIREC cohort may have identified a stronger association (92% vs. 36% increase) between fluoridated water and fluoride concentrations than we found in NHANES because of differences between plasma and urinary fluoride biomarkers, although both biomarkers reflect a mix of chronic and recent exposure [22]. The studies also differed in measurement of municipal fluoridation level (MIREC) vs. water fluoridation in home tap water (NHANES). Because measuring water fluoride in tap water samples accounts for home filtration and other between-home differences within a municipality, we would have expected this to result in a stronger association in NHANES, which was not the case. Regardless, our study replicates the findings of the MIREC study in the U.S. and builds on the MIREC study by examining the dose-response for fluoridated water intake on fluoride biomarker concentrations, finding a linear dose response relationship.

We also found strong linear associations between the amount of green/black tea consumption and plasma fluoride concentrations. Tea plants preferentially uptake or hyperaccumulate fluoride from the soil [9], and numerous studies have reported high fluoride concentrations in prepared green/black tea [18,24,28], including tea prepared with distilled (i.e., not fluoridated) water [29]. Fewer studies have related tea intake to markers of fluoride intake in humans. In the MIREC cohort, mothers who reported black tea consumption had higher urinary fluoride concentrations than mothers who did not [27], with black tea accounting for a similar amount of variance (5%) in fluoride concentrations as reported here. In Asia, where consumption of high-fluoride brick tea is prevalent, tea consumption is associated with dental fluorosis in children [30,31,32,33] and skeletal fluorosis in adults [31]. Like Till et al., we observed a stronger association with plasma fluoride for black vs. green tea, consistent with observations of higher fluoride concentrations in black vs. green tea [24], and possibly resulting from the oxidation of black tea during processing or the use of different tea plant varieties [18]. We observed a 6% increase in plasma fluoride concentrations associated with intake of other tea (i.e., tea/lemonade and herbal tea), which may be due to the presence of green/black tea in some of these products.

We extended our investigation to foods and oral health behaviors not investigated in other studies of fluoride biomarkers [26,27]. We found that other culprit foods, including grapes, coffee, fruit juice, shellfish, and processed chicken were not associated with plasma fluoride concentrations. One possible explanation was that we lacked data on whether these foods were manufactured using fluoridated water [7] or if grapes or other fruit were treated with cryolite [10]. The differences in these or other factors may have resulted in meaningful heterogeneity in the fluoride content for a given food [29,34]. Additionally, the fluoride found in food may be less bioavailable than fluoride in tea [8,34].

We observed that current use of prescription fluoride supplements was associated with a non-statistically significant decrease in plasma fluoride concentrations. Presumably, this counterintuitive result is due to the use of prescription fluoride supplements in low tap water fluoride households, which is the current recommendation of the American Dental Association [35]. Despite the frequent ingestion of toothpaste by children [36], particularly at younger ages, frequency of tooth brushing and amount of toothpaste used were not associated with plasma fluoride concentrations. Other investigators reported no association between tooth brushing frequency or toothpaste ingestion and dental fluorosis [37,38].

Our finding of a strong association of intake of fluoridated tap water and black or green tea with plasma fluoride in children and adolescents has considerable public health significance. Fluoride has been described as a “presumptive neurotoxicant”, with possible neurotoxic effects resulting from exposure in childhood [4]. No studies have related plasma fluoride concentrations to neurodevelopmental outcomes; however, plasma fluoride is positively correlated with urinary fluoride [21], which has been associated with adverse neurodevelopmental outcomes. In the MIREC cohort, each 1 mg/L increase in water fluoride concentration was associated with a reduction of 8.8 IQ points in formula-fed, but not exclusively breastfed children, suggesting that the fluoride content of water used to reconstitute formula may impact the developing brain during infancy [4]. Whether fluoride intake in later childhood could influence IQ is unknown. Evidence also suggests that prenatal fluoride exposure may have neurotoxic effects [2,3,5,6]. We observed a 64% increase in plasma fluoride associated with green/black tea consumption in adolescent females (16–19 years). Our findings therefore support the recommendation that people who want to reduce fluoride intake should limit their consumption of green and black tea.

Only 16% of our study population reported recent consumption of fluoridated tap water, which may have limited our study’s findings. First, 29% of our study population had fluoridated home tap water compared to 62% of homes sourced by municipal fluoridated water in the U.S. overall, as reported by states to the CDC Water Fluoridation Reporting System [39]. This discrepancy may result from a decrease in fluoridation between the municipal water source and delivery to the home tap. However, when we relaxed the definition of fluoridated water to ≥0.5 mg/L (resulting in 51% of our population having fluoridated tap water), we found a similar magnitude of the association of fluoridated water consumption with plasma fluoride. Additionally, due to the relatively small number of locations where enrollment occurred, NHANES participants may have been disproportionately sampled from communities with non-fluoridated water; however, we lack data to test this explanation. Second, the percentage of participants consuming fluoridated tap water was further reduced because only 54% of participants recently consumed tap water, reducing statistical power to test for an association with plasma fluoride concentrations. Our study was further limited by our lack of data on water fluoride concentrations for tap water consumed outside of the home or for bottled water, which likely resulted in an underassessment of fluoride intake from drinking water as a whole.

Our study also has several unique strengths, including a high degree of generalizability to U.S. children and adolescents. Also, we characterized recent diet using an extensive and validated dietary assessment, and categorized foods using CDC WWEIA categories. Finally, we had individual measures of home tap water (as opposed to water fluoridation levels reported by municipalities), allowing us to account for possible heterogeneity between homes within a municipality due to the use of home water filters [40] or other reasons.

## 5. Conclusions

In a population of 6–19-year-old U.S. children, we found recent intake of green or black tea and fluoridated tap water to be strongly associated with plasma fluoride. Given the relatively high prevalence of tea consumption in this population, regulatory agencies should consider fluoride intake from green or black tea consumption when developing thresholds for the optimal amount of water fluoridation. In addition, populations with higher plasma fluoride concentrations (e.g., young children and boys) may want to consider limiting green and black tea intake. Finally, the ongoing monitoring of teas sold in the U.S. to identify teas with very high or low fluoride concentrations would improve recommendations to consumers, particularly those with high tea intake.

## Figures and Tables

**Table 1:** Demographic characteristics and dietary and behavioral factors of 6–19-year-old participants (n = 3928), National Health and Nutrition Examination Survey, 2013–2016.

	Unweighted n (Weighted %)
Overall	3928 (100)
**Demographic Characteristics**	
Age categories	
6 to <9 years	854 (20)
9 to <12 years	870 (21)
12 to <15 years	853 (21)
15 to 19 years	1351 (38)
Boys	2001 (52)
Race/ethnicity ^1^	
Non-Hispanic white	1035 (51)
Hispanic	1414 (26)
Non-Hispanic Black	908 (14)
Asian	338 (5)
Other	233 (5)
Income to poverty ratio, FPL, %	
<100	1202 (23)
100 to <200	1026 (23)
≥200	1700 (54)
**Drinking Water Characteristics**	
Tap water source ^1^	
Don’t drink tap water	577 (12)
Community supply	2659 (71)
Well/cistern	245 (9)
Don’t know	383 (8)
Spring	64 (1)
Recent consumption of any home tap water	1820 (54)
Recent consumption of fluoridated home tap water (≥0.7 mg/L)	560 (16)
**Recent Diet**	
Black or green tea beverages, hot or iced	503 (13)
Other tea beverages, including herbal	36 (1)
Fruit juice	1021 (24)
Grapes/raisins	232 (6)
Shellfish	92 (2)
Coffee	219 (6)
Chicken nuggets, tenders, and patties	482 (12)
**Oral Health Behaviors**	
Recent dentist visit (<6 months)	2457 (64)
Amount of toothpaste used ^1^	
Smear	125 (4)
Pea size	824 (24)
Half load	1175 (30)
Full load	1754 (43)
Frequency of tooth brushing, times per day ^1^	
1	1210 (33)
2	2353 (62)
3	241 (5)
≥4	25 (1)
Current use of prescription fluoride drops or tablet ^2^	44 (2)

^1^ Percentages do not sum to 100 due to rounding. Percentages are population-weighted and thus cannot be directly calculated from the unweighted numbers in the table. ^2^ 6–15-year olds. Abbreviation: FPL, federal poverty level. Missing data: recent dentist, n = 11; brushing frequency, n = 99; amount of toothpaste, n = 50.

## Appendix A

**Table A1:** Percent change in plasma fluoride by demographic characteristics and modifiable sources of fluoride consumption among 6–19-year-old National Health and Nutrition Examination Survey participants from 2013–2016.

	Percent Change (95% CI) in Plasma Fluoride	
Characteristics	Unadjusted	Adjusted for Demographics, Fluoridated Tap Water Consumption, and Green/Black Tea Consumption ^1^
**Demographic Characteristics**		
Age categories		
6 to <9 years	Reference	Reference
9 to <12 years	−5.1% (−11.5, 1.8)	−7.7% (−14.4, 0.6)
12 to <15 years	−13.3% (−18.0, −8.3)	−16.9% (−21.4, −12.2)
15 to <19 years	−7.9% (−15.0, −0.2)	−11.5% (−16.8, −5.8)
Sex		
Girls	Reference	Reference
Boys	5.8% (1.6, 10.1)	5.1% (1.2, 9.1)
Race/ethnicity		
Non-Hispanic white	Reference	Reference
Hispanic	−12.3% (−22.1, −1.4)	−10.3% (−19.6, −0.1)
Non-Hispanic Black	−4.0% (−14.5, 7.7)	−2.3% (−12.4, 9.0)
Asian	−10.8% (−22.4, 2.5)	−11.2% (−22.3, 1.4)
Other	−8.3% (−18.4, 3.1)	−9.4% (−19.9, 2.3)
Income to poverty ratio, FPL, %		
<100	Reference	Reference
100 to <200	2.9% (−3.3, 9.4)	1.4% (−3.9, 6.9)
≥200	3.9% (−5.3, 14.0)	−0.8% (−8.2, 7.3)
**Drinking Water Characteristics**		
Tap water source		
Don’t drink tap water	Reference	Reference
Community supply	8.4% (−1.1, 18.8)	1.8% (−6.1, 10.3)
Well/cistern	10.3% (−7.0, 30.9)	4.1% (−11.0, 21.8)
Don’t know	1.7% (−11.3, 16.8)	−6.0% (−17.8, 7.6)
Spring	−8.2% (−30.1, 20.6)	−8.8% (−31.4, 21.4)
Recent consumption of fluoridated home tap water (≥0.7 mg/L)		
No	Reference	Reference
Yes	34.8% (22.0, 49.0)	35.5% (22.0, 50.5)
**Recent Diet**		
Green/black tea		
No consumption	Reference	Reference
Any consumption	37.7% (22.1, 55.2)	41.7% (26.7, 58.4)
Other tea		
No consumption	Reference	Reference
Any consumption	0.5% (−22.7, 30.7)	6.4% (−18.2, 38.4)
Fruit juice		
No consumption	Reference	Reference
Any consumption	−0.1% (−6.1, 6.3)	0.6% (−4.4, 6.0)
Grapes/raisins		
No consumption	Reference	Reference
Any consumption	7.9% (−1.7, 18.4)	4.1% (−3.5, 12.3)
Shellfish		
No consumption	Reference	Reference
Any consumption	−2.9% (−11.4, 6.3)	3.2% (−5.9, 13.2)
Coffee beverages		
No consumption	Reference	Reference
Any consumption	4.8% (−6.7, 17.7)	7.8% (−3.2, 20.0)
Chicken nuggets, tenders, and patties		
No consumption	Reference	Reference
Any consumption	4.4% (−3.2, 12.7)	5.1% (−2.3, 13.0)
**Oral Health**		
Recent dentist visit (<6 months)		
No	Reference	Reference
Yes	1.0% (−5.5, 7.9)	−0.7% (−6.5, 5.5)
Amount of toothpaste used		
Smear	Reference	Reference
Pea size	−2.5% (−14.9, 11.7)	0.0% (−12.6, 14.4)
Half load	−4.5% (−16.2, 8.8)	−1.3% (−12.4, 11.1)
Full load	−4.6% (−17.2, 9.9)	−0.7% (−13.7, 14.1)
Frequency of tooth brushing, times per day		
1	Reference	Reference
2	−1.4% (−6.5, 4.0)	1.3% (−3.6, 6.4)
3	3.0% (−8.0, 15.2)	9.1% (0.0, 18.9)
≥4	−3.1% (−19.5, 16.6)	7.0% (−13.5, 32.3)
Current use of fluoride drops or tablets ^2^		
No	Reference	Reference
Yes	−13.7% (−29.7, 5.9)	−12.2% (−32.0, 13.4)

^1^ Adjusted for age, sex, race/ethnicity and with mutual adjustment for recent consumption of fluoridated tap water and green/black tea. ^2^ 6–15 year olds.

**Table A2:** Characteristics of 6–19 year old respondents, by recent green/black tea consumption, National Health and Nutrition Examination Survey, 2013–2016.

	Unweighted n (Weighted %)	
Characteristics	Recent Black/Green Tea Consumption (n = 503)	No Recent Black/Green Tea Consumption (n = 3425)
**Demographic Characteristics**		
Age categories		
6 to <9 years	73 (12)	781 (21)
9 to <12 years	99 (17)	771 (21)
12 to <15 years	117 (23)	736 (21)
15 to <19 years	214 (48)	1137 (37)
Sex		
Girls	263 (51)	1664 (47)
Boys	240 (49)	1761 (53)
Race/ethnicity ^1^		
Non-Hispanic white	148 (52)	887 (50)
Hispanic	173 (25)	1241 (26)
Non-Hispanic Black	100 (11)	808 (15)
Asian	51 (5)	287 (5)
Other	31 (7)	202 (5)
Income to poverty ratio, FPL, % ^1^		
<100	154 (22)	1048 (23)
100 to <200	119 (26)	907 (23)
≥200	230 (53)	1470 (54)
**Drinking Water Characteristics**		
Tap water source ^1^		
Don’t drink tap water	74 (11)	503 (12)
Community supply	314 (65)	2345 (72)
Well/cistern	52 (13)	193 (8)
Don’t know	57 (11)	326 (8)
Spring	6 (1)	58 (1)
Recent consumption of fluoridated home tap water (≥7 mg/L)		
No	454 (88)	2914 (84)
Yes	49 (12)	511 (16)
**Recent Diet**		
Other tea		
No recent consumption	498 (100)	3394 (99)
Any recent consumption	5 (0)	31 (1)
Fruit juice		
No recent consumption	401 (81)	2506 (75)
Any recent consumption	102 (19)	919 (25)
Grapes/raisins		
No recent consumption	483 (96)	3213 (94)
Any recent consumption	20 (4)	212 (6)
Shellfish		
No recent consumption	488 (98)	3348 (98)
Any recent consumption	15 (2)	77 (2)
Coffee beverages		
No recent consumption	470 (94)	3239 (94)
Any recent consumption	33 (6)	186 (6)
Chicken nuggets, tenders, and patties		
No recent consumption	439 (85)	3007 (88)
Any recent consumption	64 (15)	418 (12)
**Oral Health**		
Recent dentist visit (<6 months)		
No	214 (43)	1246 (35)
Yes	286 (57)	2171 (65)
Amount of toothpaste used		
Smear	11 (3)	114 (4)
Pea size	82 (17)	742 (25)
Half load	147 (32)	1028 (29)
Full load	258 (48)	1496 (42)
Frequency of toothbrushing, times per day ^1^		
1	170 (32)	1040 (33)
2	289 (62)	2064 (62)
3	32 (5)	209 (5)
≥4	2 (0)	23 (1)
Current use of prescription fluoride drops or tablet ^2^		
No	314 (98)	2463 (98)
Yes	4 (2)	40 (2)

^1^ Percentages do no sum to 100 due to rounding. Percentages are population-weighted and thus cannot be directly calculated from the unweighted numbers in the table. ^2^ 6–15 year olds. Abbreviation: FPL, federal poverty level.
